# Glutathione homeostasis is significantly altered by quercetin via the Keap1/Nrf2 and MAPK signaling pathways in rats

**DOI:** 10.3164/jcbn.17-40

**Published:** 2017-12-12

**Authors:** Weina Gao, Lingling Pu, Ming Chen, Jingyu Wei, Zhonghao Xin, Yawen Wang, Zhanxin Yao, Tala Shi, Changjiang Guo

**Affiliations:** 1 Tianjin Institute of Health and Environmental Medicine, NO.1 Dali Road, Tianjin 300050, P. R. China; 2 The People’s Hospital of Lichuan, Jiangxi Province, 344600, P. R. China

**Keywords:** quercetin, glutathione, glutamate cysteine ligase, Nrf2, MAPKs

## Abstract

Previously, we showed that 0.5% quercetin simultaneously decreased serum homocysteine and glutathione (GSH) levels in rats. The aim of the present study was to investigate the effects of 0.5% quercetin on GSH metabolism, related enzymes and signal pathways in rats. Rats were fed the control diet and 0.5% quercetin-supplemented diet for 6 weeks. The results showed that quercetin reduced serum and hepatic content of GSH and the ratio of GSH and oxidized glutathione (GSSG), enhanced hepatic activity and mRNA expression of glutathione *S*-transferase (GST), inhibited hepatic activity and mRNA expression of glutamate cysteine ligase (GCL), and decreased hepatic glutathione reductase (GR) mRNA expression. Levels of phosphorylated p38 and extracellular signal-regulated kinase (ERK) 1/2 mitogen-activated protein kinases (MAPKs) increased, while that of nuclear factor E2-like 2 (Nrf2) protein decreased after quercetin treatment. However, no significant hepatotoxicity was noted. We concluded that quercetin treatment altered hepatic GSH metabolism by modulating GSH metabolic enzyme activities and mRNA expression in rats, and p38, ERK1/2 MAPKs, and Nrf2 were involved in modulating GSH metabolism-related enzymes.

## Introduction

**Fig. 1:**
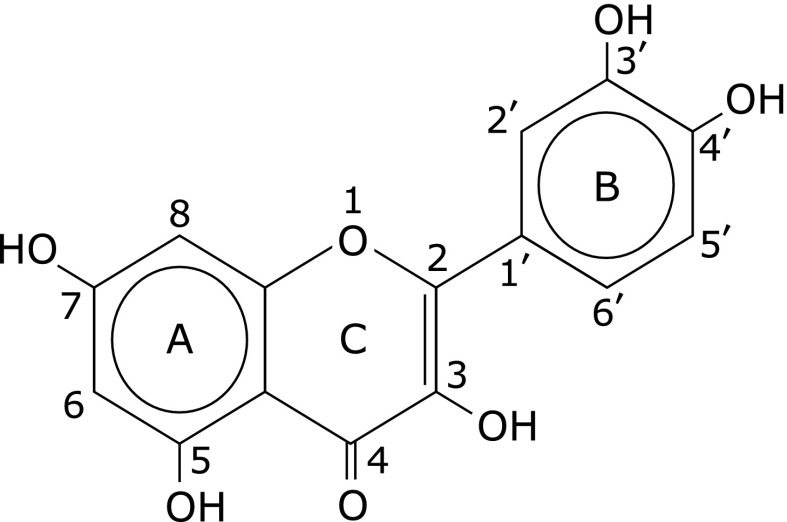
Chemical structure of quercetin.

Quercetin (3,3',4',5,7-pentahydroxyflavone) is one of the common dietary polyphenols present in fruits, vegetables, beverages, and teas,^(1–4)^ as well as in various medicinal herbs (Fig. 1).^(5,6)^ Quercetin has both medicinal and nutritional values such as antioxidant, anti-inflammatory, immuno-stimulatory, and anti-chronic-disease properties.^(7,8)^ Previously, we fed rats with diets containing 1% methionine in combination with 0.1%, 0.5% and 2.5% quercetin. It was found that 0.5% and 2.5% quercetin displayed a significant impact on glutathione (GSH) homeostasis, in which hepatic level of GSH was notably decreased. A significant hepatotoxic action was demonstrated when 2.5% quercetin was supplied.^(9)^ Chen *et al.*^(10)^ also found that 0.05% quercetin decreased serum GSH content, and increased GSH-Px activity in rats. However, the underlying mechanisms are not well clarified yet.

**Fig. 2:**
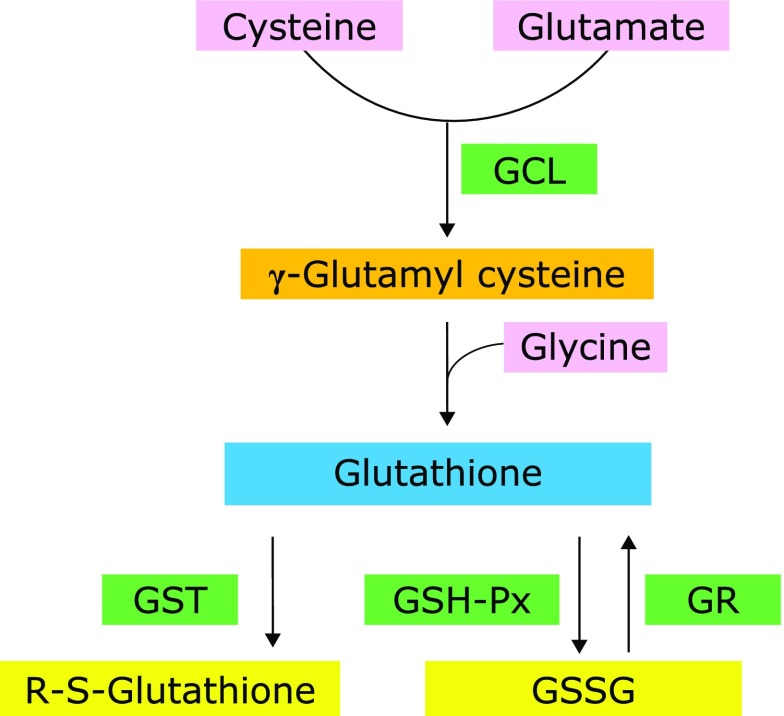
Glutathione metabolic pathways. GCL, glutamate cysteine ligase; GST, glutathione *S*-transferase; GSH-Px, glutathione peroxidase; GR, glutathione reductase; GSSG, oxidized glutathione.

GSH, a tripeptide composed of glycine, cysteine, and glutamate, is the most abundant antioxidant *in vivo* and considered one of the most important determinants of the antioxidant defense system.^(11)^ GSH quenches reactive hydroxyl free radicals and oxygen-centered free radicals by providing the protons for the antioxidant enzymes or conjugates with endogenous substances and xenobiotics by the reactions catalyzed by detoxication enzymes such as glutathione *S*-transferase (GST).^(12,13)^ Glutathione peroxidase (GSH-Px) catalyzes reductive processes for lipid hydroperoxides, using GSH as an electron donor.^(14)^ GST catalyzes the reaction of GSH with endogenous substances or diverse xenobiotics by forming mixed disulfides.^(15)^ In those processes, GSH itself is converted to its oxidative form GSSG, which is regenerated to two GSH molecules by glutathione reductase (GR). Glutamate cysteine ligase (GCL), also known as γ-glutamylcysteine synthetase, catalyzes the first rate-limiting enzymatic step in GSH synthesis.^(16)^ Among the four enzymes participating in GSH metabolism, GSH-Px and GST consume GSH, while GR and GCL are responsible for GSH regeneration and synthesis, respectively (Fig. 2).

Two signaling pathways play critical roles in modulating the expression of GSH metabolic enzymes. Kelch-like erythroid cell-derived-associated protein 1 (Keap1)/nuclear factor E2-like 2 (Nrf2) is responsible for transcriptional activation of a large number of genes that are regulated by antioxidant response elements in their promoters. The Keap1/Nrf2 signal pathway has been demonstrated to play a role in regulating the expression of GSH metabolic enzymes.^(17,18)^ Mitogen-activated protein kinases (MAPKs) comprise a family of signaling proteins that convert extracellular stimuli into intracellular transduction pathways via phosphorylation of a cascade of substrates. The MAPK signal pathway is also involved in the regulation of GSH metabolism.^(19)^

The liver plays a critical role in metabolic processes and is the principal detoxifying organ involved in the clearance of toxic chemicals and metabolic waste products from the body.^(20–22)^ The liver also serves as an important organ in GSH metabolism and redox reactions depending partially on GSH availability.^(15,16)^ We hypothesize that quercetin at high intake levels alters GSH homeostasis in the liver through modulating related signaling pathways. To test this hypothesis, we investigated the effects of 0.5% quercetin on hepatic GSH metabolic enzymes and explored the changes in the Keap1/Nrf2 and MAPKs pathways.

## Materials and Methods

### Animals, diets, and experimental protocol

Animal handling was performed according to the current Chinese legislation on the care and use of laboratory animals. The experimental protocol was approved by the Ethical Committee of the Department of Scientific Management of the institute. Twenty male Wistar rats, weighing 180–200 g, were purchased from the Laboratory Animal Center, Chinese Academy of Military Medical Sciences (Beijing, China) and housed individually in stainless-steel cages. The room temperature was between 22 and 24°C, and relative humidity between 40% and 60%, with 12 h light/dark cycles. Food and tap water were provided ad libitum. Dietary intake was recorded every day, and body weight once a week. After being acclimatized on a polyphenol-free semisynthetic diet (AIN-93 formula) for 5 days,^(23)^ the rats were divided randomly into two groups based on body weight and maintained on the control diet (AIN-93 diet) and 0.5% quercetin (Sigma-Aldrich, St. Louis, MO) supplemented AIN-93 diet (0.5%Q), respectively, for 6 weeks. At the end of the experiment, all rats were fasted overnight, and blood samples were collected from the orbital plexus under ether anesthetization. The serum was separated and stored at −80°C. Liver tissues were sampled immediately, cleaned up in ice-cold saline, and frozen at −80°C until use.

### Contents of quercetin and its methylated metabolites in the serum and liver

Quercetin and its methylated metabolites in the serum and liver were determined after enzymatic deconjugation by a high performance liquid chromatography (HPLC) procedure described previously by Chen *et al.*^(24)^

### Serum activities of alanine aminotransferase and aspartate aminotransferase

Serum alanine aminotransferase (ALT) and aspartate aminotransferase (AST) activities were detected using commercial kits purchased from the Biosino Bio-Technology and Science Inc. (Beijing, P.R. China).

### Hepatic contents of GSH, GSSG, and malondialdehyde

The contents of hepatic GSH and GSSG were measured using assay kits purchased from Nanjing Jiancheng Bioengineering Institute (Nanjing, Jiangsu). Malondialdehyde (MDA) was assayed spectrophotometrically by the reaction with thiobarbituric acid.^(25)^ Protein concentration was determined using a bicinchonininc acid (BCA) reagent kit (Sangon Biotech, Shanghai, China).

### Hepatic activities of GSH-Px, GST, GR and GCL

 Activities of hepatic GSH-Px, GST, GR and GCL were assayed by commercial kits purchased from Nanjing Jiancheng Bioengineering Institute (Nanjing, China). The kits numbers of GSH-Px, GST, GR and GCL were A005, A004, A062 and A120, respectively. All measurements were performed strictly in line with the manufacturer’s instructions.

### Quantitative real-time polymerase chain reaction analysis

Hepatic mRNA expressions of GSH metabolic enzymes and extracellular signal-regulated kinase (ERK), p38, and c-Jun N-terminal kinase (JNK) were determined by quantitative real-time polymerase chain reaction (qPCR).^(26)^ Total hepatic RNA was extracted using the TRIzol reagent (Roche, Basel, Switzerland). The first cDNA strand was synthesized using a cDNA Synthesis kit (Roche). qPCR was performed after cDNA synthesis in a FastStart Universal SYBR Green Master mix (Roche). The primers used for the genes studied are listed in Table 1. Finally, melting curve analysis was performed by slowly cooling the PCR mixture with simultaneous measurement of the SYBR Green I signal intensity by Takara PCR Thermal Cycler Dice Real Time System (Takara TP800; Takara, Tokyo, Japan). The Δ threshold cycle (Ct) method was used to evaluate the relative quantification, and β-actin and glyceraldehyde 3-phosphate dehydrogenase (GAPDH) as reference.

### Western blotting analysis

Hepatic protein expression of Keap1, Nrf2, ERK1/2, phosphorylated-ERK (p-ERK), p38, p-p38, JNK and p-JNK were analyzed by western blotting.^(26)^ The software Image-Pro Plus 6 (Rockville, MD) was used to analyze the protein bands, and the results were determined by the ratio of target protein density to that of control after being corrected by GAPDH. Anti-JNK, anti-p-JNK, anti-p38, anti-p-p38, anti-ERK1/2, anti-p-ERK1/2 and anti-Keap1 were purchased from Cell Signaling Technology Ltd. (Boston, MA), anti-Nrf2 from Abcam Ltd. (Cambridge, UK), anti-GAPDH from KangChen Ltd. (Shanghai, China), horseradish peroxidase (HRP)-labeled goat anti-mouse IgG and HRP-labeled goat anti-rabbit IgG from Bomeike Bio Ltd. (Beijing, China), and HRP-labeled donkey anti-goat IgG from Santa Cruz Biotechnology (Santa Cruz, CA).

### Statistical analysis

Data are presented as means ± SD. The statistical analysis was performed using the SPSS 10.01 software (SPSS Inc., Chicago, IL). Student’s *t* test was performed to compare the differences between the control and the quercetin supplemented group. Differences between two groups were considered statistically significant at *p*<0.05.

## Results

### Dietary intake and body weight

The rats fed the quercetin-supplemented diet were similar to those in the control group in both food intake and body weight gain, indicating that 0.5% quercetin supplementation had no effect on dietary consumption and growth (data not shown).

### Quercetin and its methylated metabolites

No peaks corresponding to quercetin aglycone or its methylated metabolites were detected on HPLC chromatograms for the serum and liver samples from the control rats. On the other hand, distinct peaks corresponding to quercetin aglycone, tamarixetin, and isorhamnetin were identified in the quercetin-supplemented group. The serum contents of quercetin aglycone, tamarixetin, and isorhamnetin were 52.32 ± 11.27, 253.58 ± 37.54, and 253.18 ± 37.48 µmol/L, and hepatic contents were 22.55 ± 3.46, 36.42 ± 4.21, and 20.21 ± 2.33 nmol/g protein, respectively.

### Serum ALT and AST activity, and hepatic lipid peroxidation

**Fig. 3:**
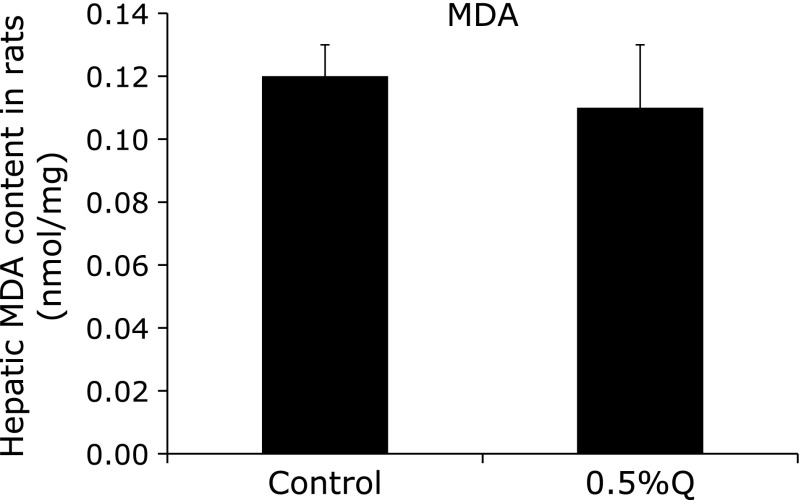
Hepatic malondialdehyde (MDA) content in rats. MDA, malondialdehyde; 0.5%Q, 0.5% quercetin-supplemented group. Results are expressed as mean and standard deviation (*n* = 10).

**Fig. 4:**
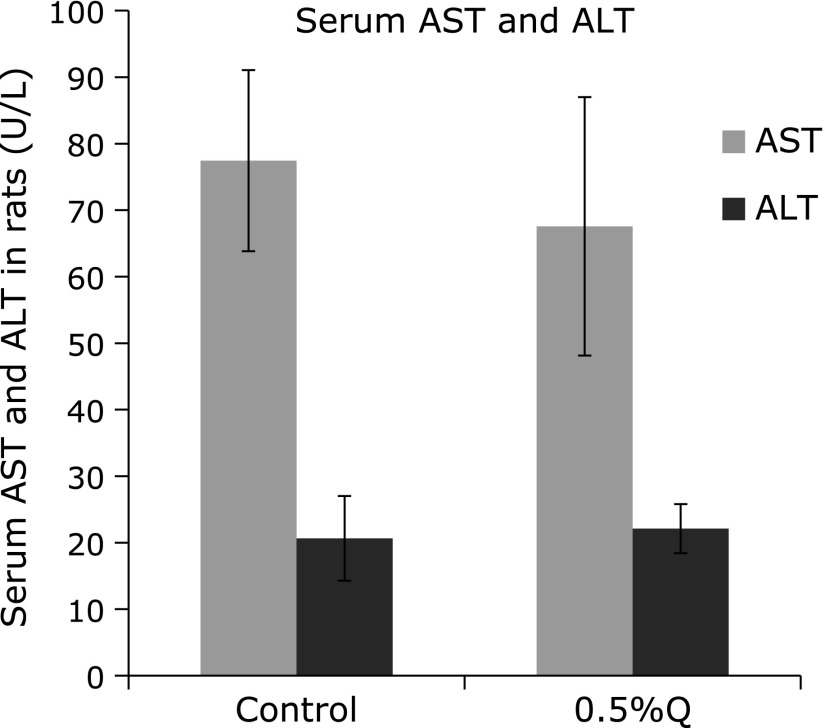
Serum activities of aspartate aminotransferase (ALT) and alanine aminotransferase (AST) in rats. AST, aspartate aminotransferase; ALT, alanine aminotransferase; 0.5%Q, 0.5% quercetin-supplemented group. Results are expressed as mean and standard deviation (*n* = 10).

There was no significant difference between the control and the quercetin-supplemented group in hepatic MDA content and serum ALT and AST activities, suggesting that no significant hepatotoxicity was detected after 0.5% quercetin supplementation (Fig. 3 and 4).

### Serum and hepatic GSH, GSSG, and ratio of GSH/GSSG

The serum content of GSH and the ratio of GSH/GSSG decreased significantly after 0.5% quercetin supplementation compared to that in the control (*p*<0.05). The hepatic contents of GSH and GSSG and the ratio of GSH/GSSG also decreased significantly in the quercetin-supplemented group (*p*<0.05) (Table 2). Hepatic GSH was reduced by 48.7% and GSSG by 10.5%, indicating that hepatic GSH content declined more than GSSG content in response to the quercetin treatment.

### Hepatic mRNA expression and activity of GSH-Px, GST, GCL and GR

An increase in GSH-Px activity and a decrease in GR activity in the liver were noted in the quercetin-supplemented rats but without statistical significance (Table 3). Hepatic activity and mRNA expression of GST significantly increased, whereas GCL expression significantly decreased in rats fed the quercetin-supplemented diet compared to that in rats fed the control diet (*p*<0.05). Hepatic mRNA expression of GR was significantly inhibited after quercetin treatment (*p*<0.05) (Table 4).

### Keap1 and Nrf2 protein expression

**Fig. 5:**
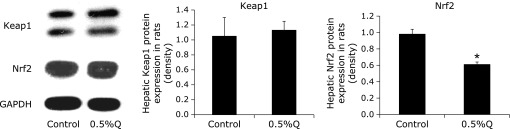
Hepatic Kelch-like erythroid cell-derived-associated protein 1 (Keap1) and nuclear factor E2-like 2 (Nrf2) protein expression in rat liver (*n* = 3, *x̄* ± s). *Significantly different from the control group.

The hepatic protein expression of Keap1 was slightly increased in the 0.5% quercetin-supplemented group, whereas Nrf2 level was significantly lower in the 0.5% quercetin-supplemented group than in the control group (*p*<0.05) (Fig. 5).

### Hepatic ERK, p38, and JNK mRNA and protein expression

**Fig. 6:**
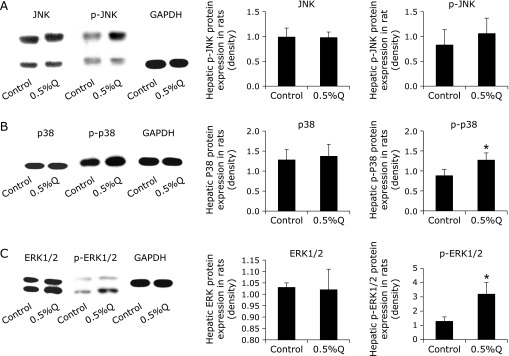
Hepatic protein expression of MAPKs. (A) Hepatic c-Jun N-terminal kinase (JNK) and phosphorylated-JNK (p-JNK); (B) hepatic p38 and phosphorylated p38 (p-p38) protein expression in rat liver; (C) hepatic extracellular signal-regulated kinase (ERK)1/2 and phosphorylated-ERK1/2 (p-ERK1/2) (*n* = 3, *x̄* ± s). Con: control; 0.5%Q: 0.5% quercetin group. *Significantly different from the control group.

Hepatic p-p38 and p-ERK protein expressions were increased significantly in the 0.5% quercetin-supplemented group. Hepatic p-JNK level was also increased but no statistical difference was detected. No remarkable change in JNK, p38, and ERK mRNA and protein expressions in the liver was found after quercetin supplementation (Table 5, Fig. 6).

## Discussion

In our experiment, hepatic GSH content was significant decreased in rats treated by 0.5% quercetin. This is consistent with the data reported by Choi *et al.*^(27,28)^ Metodiewa *et al.*^(29)^ showed that quercetin altered GSH homeostasis directly. The C4-keto moiety and C2=C3 double bond in quercetin structure facilitated the formation of *o*-quinoid type metabolites such as quinones and quinone methides, in which quercetin initially is oxidized to an *o*-quinone, which is rapidly isomerized to quinone methides. ^(30–32)^ These quinoids are trapped by GSH, and isomeric mixtures of quinoid GSH conjugates are formed. Simultaneously, a decrease in GSH content occurs.^(33)^ However, some studies showed that quercetin could impact GSH homeostasis via other mechanisms. For example, Choi *et al.*^(27)^ demonstrated that quercetin treatment reduced hepatic GR activity by 34% in rats, which also contributed to the decline of GSH content.

In the present study, we confirmed that 0.5% quercetin treatment induced a significant decrease in the contents of hepatic GSH and GSSG, as well as the ratio of GSH/GSSG. In addition, the decline of GSH level was more significant than that of GSSG. We further investigated the enzymatic activity and mRNA expression of GCL. The results showed that hepatic activity and mRNA expression of GCL were decreased after exposure to quercetin, indicating that GSH synthesis was suppressed. We also found that mRNA expression of GR was significantly reduced in response to quercetin treatment. Since GCL and GR are responsible for GSH synthesis and regeneration, the inhibition of those two enzymes will contribut to the decrease of hepatic GSH level after quercetin treatment. We also showed that hepatic GST activity and mRNA expression were enhanced after quercetin treatment. Similar results were reported by others,^(34–38)^ showing that the administration of quercetin increased the activity of GST in rats, silkworms, and cultured cells. Since GST catalyzes the conjugation of GSH with endogenous compounds or xenobiotics, increased GST activity will require more GSH as a substrate, leading to the consumption of hepatic GSH.

Evidences have showed that GSH depletion was accompanied with oxidative stress even without significant oxidative stimulation. Vaziri *et al.*^(39)^ found a 3-fold decrease in tissue GSH content after exposure to l-buthionine sulphoximine (BSO), a selective inhibitor of gamma glutamyl cysteine synthetase, and oxidative stress was resulted in Sprague-Dawley rats. Beddowes *et al.*^(40)^ demonstrated that 8-oxodeoxyguanosine and malondialdehyde deoxyguanosine adducts were significantly increased in HepG2 cells after being treated by BSO to deplete GSH. Armstrong *et al.*^(41)^ showed that after BSO treatment, there was an early decline in cellular GSH, followed by an increase in reactive oxygen species production in human B lymphoma cell line. Besides BSO, other reagents such as vinylidene chloride, diethylmaleate and doxycycline are also used to induce GSH depletion and oxidative stress is resulted. For example, incubations of liver homogenates from glutathione-depleted mice and rats after exposure to vinylidene chloride and diethylmaleate resulted spontaneously in a large increase of the lipid peroxidation.^(42)^ Down-regulation of glutamyl-cysteine synthetase gene expression by doxycycline resulted in a reduction in mitochondrial GSH levels, which led to increased oxidative stress in PC12 cells.^(43)^ In previous experiments, we found that serum MDA was significantly increased in rats treated by 0.5%–2.5% quercetin for 6 weeks.^(9,10)^ However, in the present study, we do not find significant changes in hepatic MDA level after 0.5% quercetin administration, although serum and hepatic GSH content was distinctly decreased. Thereby, we think that 0.5% quercetin may be a critical level in inducing a pro-oxidant effect. Serum ALT and AST levels also did not increase significantly in response to quercetin treatment. Thereby, 0.5% quercetin supplementation did not induce a significant toxic action in the liver. Previously, it was demonstrated that the administration of quercetin markedly increased the amount of α-tocopherol in rat plasma and liver.^(27,44)^ The adverse effects of quercetin on GSH homeostasis may be compensated by increased α-tocopherol level, because α-tocopherol is also one of the critical antioxidants *in vivo*. However, the content of hepatic α-tocopherol was not measured in this study. It should be mentioned that quercetin is extensively metabolized after absorption,^(45,46)^ which may limit the action of quercetin on GSH metabolism. In this study, we detected the contents of quercetin and its methylated derivatives in the serum and liver. The results confirmed that quercetin was present largely in the form of its methylated derivatives in the serum and liver. Although quercetin metabolites is relatively weaker in antioxidant capacity,^(47)^ it have been demonstrated that part of quercetin metabolites could be transformed to quercetin aglycon and play an antioxidant role in tissues.^(48,49)^ Therefore, it is possible that quercetin and its metabolites in the tissues can play an antioxidant role and make up partly for the decline of GSH content.

Since the GSH homeostasis is tightly controlled by several upstream signaling pathways, we further investigated the change in the Keap1/Nrf2 and MAPKs pathways. Nrf2 expression plays an important role in modulating the expression of some GSH-related enzymes, especially GCL and GR.^(50,51)^ Nrf2 down-expression blocked the GCL expression induced by isorhamnetin.^(52)^ Down-expression of Nrf2 also decreased GR activity and mRNA expression, and reduced GSH content in fish gill with amino acid phenylalanine deficiency.^(53)^ Moreover, Nrf2 knockout led to a significantly reduction in hepatic GST expression of mRNA, protein, and catalytic activity in mice.^(54)^ In this study, we found that hepatic Nrf2 protein expression was significantly decreased, which was correlated with decreased activities and mRNA expressions of GCL and GR in 0.5% quercetin-treatment group. Thereby, it is likely that down-regulated Keap1/Nrf2 signaling pathway contributes to the change in GSH homeostasis after quercetin exposure. The mammalian MAPK family consists of ERK1/2, p38 and JNK.^(55,56)^ It had been demonstrated that quercetin increased phosphorylation of p38 and ERK1/2 in BV-2 microglial cells and induced phosphorylation of p38 and affected GSH homeostasis in HepG2 cells.^(57,58)^ Quercetin derivatives such as isorhamnetin and rutin were also effective in increasing ERK1/2 phosphorylation.^(19,52)^ In the present study, the hepatic protein expressions of p-ERK1/2 and p-p38 were increased after quercetin treatment, indicating that quercetin altered GSH homeostasis partly via MAPKs signal pathway.

## Conclusion

In conclusion, this study demonstrated that 0.5% quercetin treatment reduced hepatic GSH content by enhancing the activity and mRNA expression of GST, and inhibiting the activity and mRNA expression of GCL. This action is associated with the changes of Keap1/Nrf2 and MAPKs signal pathways. More profound changes in GSH homeostasis may be manifested when quercetin is administered at higher doses. Thereby, the safety of quercetin administration still needs to be further investigated.

## Figures and Tables

**Table 1:** Sequences of the primers used in quantitative real-time polymerase chain reaction (qPCR) analysis

Genes		Primers
GSH-Px	F	5'-AGGAGAATGGCAAGAATGAAGAGA-3'
	R	5'-GGAAGGTAAAGAGCGGGTGAG-3'
GST	F	5'-TTCTGACCTCTTTCCCTCTGCT-3'
	R	5'-CTTGAAAACCTTCCTTGCTTCTTC-3'
GR	F	5'-TCTACTCGACCGCCTTCACC-3'
	R	5'-GCCAACCACCTTCTCCTCTTT-3'
GCL	F	5'-CTGCACATCTACCACGCAGTCA-3'
	R	5'-ATCGCCGCCATTCAGTAACAA-3'
JNK	F	5'-GGATTTGGAGGAGCGAACTA-3'
	R	5'-TGACAGACGGCGAAGAGAC-3'
ERK1	F	5'-ATCTGTGATTTTGGCCTTGC-3'
	R	5'-GCCCTTGGAGTTAAGCATGA-3'
ERK2	F	5'-GCCGCGCTACACTAATCTCT-3'
	R	5'-AGGTCTGGTGCTCAAAAGGA-3'
p38	F	5'-GGCACACTGATGACGAAATG-3'
	R	5'-CCACGGACCAAATATCCACT-3'
β-Actin	F	5'-GTCCCTCACCCTCCCAAAA-3'
	R	5'-GCTGCCTCAACACCTCAACCC-3'
GAPDH	F	5'-TGATTCTACCCACGGCAAGTT-3'
	R	5'-TGATGGGTTTCCCATTGATGA-3'

GSH-Px, glutathione peroxidase; GST, glutathione *S*-transferase; GR, glutathione reductase; GCL, glutamate cysteine ligase; JNK, c-Jun NH2-terminal kinase; ERK1, extracellular signal-regulated kinase 1; ERK2, extracellular signal-regulated kinase 2; GAPDH, glyceraldehyde 3-phosphate dehydrogenase. F, forward; R, reverse.

**Table 2:** Serum and hepatic contents of glutathione (GSH) and oxidized glutathione (GSSG), and the ratio of GSH to GSSG in rats

Group	Serum				Liver		
	GSH (µmol/g prot)	GSSG (µmol/g prot)	GSH/GSSG		GSH (µmol/g prot)	GSSG (µmol/g prot)	GSH/GSSG
Control	45.62 ± 10.81	3.87 ± 0.91	12.27 ± 3.01		1.19 ± 0.23	1.91 ± 0.21	0.86 ± 0.19
0.5%Q	8.12 ± 3.23*	3.55 ± 0.68	1.75 ± 0.68*		0.61 ± 0.28*	1.71 ± 0.14*	0.47 ± 0.21*

Values are presented as mean ± standard deviation (*n* = 10). *p<0.05, compared with control. Serum GSH and GSH/GSSG, and hepatic GSH, GSSG and GSH/GSSG all decreased significantly, and and *p* value were 0.000, 0.000, 0.000, 0.023 and 0.000 respectively. GSH, glutathione; 0.5%Q, 0.5% quercetin-supplemented group.

**Table 3:** Hepatic activities of glutathione (GSH) metabolic enzymes in rats

Group	GSH-Px (U/mg prot)	GST (U/mg prot)	GR (U/g prot)	GCL (U/mg prot)
Control	847.38 ± 54.05	100.79 ± 6.72	37.91 ± 12.13	0.28 ± 0.05
0.5%Q	886.10 ± 72.40	122.25 ± 5.37*	22.84 ± 10.65	0.19 ± 0.02*

Values are presented as mean ± standard deviation (*n* = 10). *p<0.05, compared with control. Hepatic activity of GST increased, while GCL decreased significantly after administration of 0.5% quercetin, and *p* value were 0.000 and 0.002 respectively. GSH-Px, glutathione peroxidase; GST, glutathione *S*-transferase; GR, glutathione reductase; GCL, glutamate cysteine ligase; 0.5%Q, 0.5% quercetin-supplemented group.

**Table 4:** Hepatic mRNA expression of glutathione (GSH) metabolic enzymes in rats

Group	GSH-Px	GST	GR	GCL
Control	1.01 ± 0.02	0.99 ± 0.13	0.97 ± 0.06	0.91 ± 0.17
0.5%Q	1.17 ± 0.16	2.49 ± 0.25*	0.17 ± 0.06*	0.37 ± 0.06*

Values are presented as mean ± standard deviation (*n* = 3). *p<0.05, compared with control. Hepatic mRNA expression of GST increased, while GR and GCL decreased significantly after administration of 0.5% quercetin, and *p* value were 0.001, 0.000 and 0.006 respectively. GSH-Px, glutathione peroxidase; GST, glutathione *S*-transferase; GR, glutathione reductase; GCL, glutamate cysteine ligase; 0.5%Q, 0.5% quercetin-supplemented group.

**Table 5:** Hepatic mRNA expression of mitogen-activated protein kinases (MAPKs) in rats.

Group	ERK1	ERK2	JNK	p38
Control	0.88 ± 0.13	1.02 ± 0.29	1.06 ± 0.28	1.01 ± 0.11
0.5%Q	0.99 ± 0.23	0.96 ± 0.16	1.03 ± 0.49	1.01 ± 0.31

Values are presented as mean ± SD (*n* = 3). ERK1, extracellular signal-regulated kinase 1; ERK2, extracellular signal-regulated kinase 2; JNK, c-Jun N-terminal kinase.

## Abbreviations

ALT: alanine aminotransferase
AST: aspartate aminotransferase
BCA: bicinchonininc acid
BSO: l-buthionine sulphoximine
ERK1/2: extracellular signal-regulated kinase 1/2
GCL: glutamate cysteine ligase
GR: glutathione reductase
GSH: glutathione
GSH-Px: glutathione peroxidase
GST: glutathione *S*-transferase
HPLC: high performance liquid chromatography
JNK: c-Jun NH2-terminal kinase
Keap1: Kelch like ECH-associated protein 1
MAPKs: mitogen-activated protein kinases
MDA: malondialdehyde
Nrf2: nuclear factor erythroid 2-like 2
